# Application of bio-organic amendments improves soil quality and yield of fennel (*Foeniculum vulgare* Mill.) plants in saline calcareous soil

**DOI:** 10.1038/s41598-023-45780-2

**Published:** 2023-11-14

**Authors:** Omar A. A. I. Al-Elwany, Abir M. H. A. Mohamed, Ahmed S. Abdelbaky, Mohamed A. Tammam, Khaulood A. Hemida, Gehad H. S. Hassan, Mohamed T. El-Saadony, Khaled A. El-Tarabily, Synan F. AbuQamar, Taia A. Abd El-Mageed

**Affiliations:** 1 Department of Horticulture, Faculty of Agriculture, Fayoum University, Fayoum, 63514 Egypt; 2 Department of Agricultural Microbiology, Faculty of Agriculture, Fayoum University, Fayoum, 63514 Egypt; 3 Department of Biochemistry, Faculty of Agriculture, Fayoum University, Fayoum, 63514 Egypt; 4 Department of Botany, Faculty of Science, Fayoum University, Fayoum, 63514 Egypt; 5 Department of Agricultural Microbiology, Faculty of Agriculture, Zagazig University, Zagazig, 44511 Egypt; 6 Department of Biology, College of Science, United Arab Emirates University, Al Ain, 15551 United Arab Emirates; 7 Department of Soil and Water, Faculty of Agriculture, Fayoum University, Fayoum, 63514 Egypt

**Keywords:** Plant physiology, Abiotic

## Abstract

The impact of bio-organic amendments on crop production is poorly understood in saline calcareous soils. The aim in the present study was to determine the effects of the application of organic manure along with lactic acid bacteria (LAB) on soil quality, and morpho-physio-biochemical responses, seed yield (SY) and essential oil yield (EOY) of fennel plants (*Foeniculum vulgare* Mill.) grown in saline calcareous soils. Eight treatments of farmyard manure (FM) or poultry manure (PM) individually or combined with *Lactobacillus plantarum* (*Lp*) and/or *Lactococcus lactis* (*Ll*) were applied to saline calcareous soil in two growing seasons. Either FM or PM combined with LAB had beneficial effects on lowering ECe, pH and bulk density and increasing total porosity, organic matter, and water and nutrient retention capacities in addition to total bacterial population in the soil. Growth, nutrient uptake, SY and EOY of plants were also enhanced when fennel seeds were inoculated with *Lp* and/or *Ll* and the soil was amended with any of the organic manures under unfavorable conditions. Compared to control (no bio-organic amendments), FM + *Lp* + *Lt* or PM + *Lp* + *Lt* treatment signficantlly (*P* ≤ 0.05) increased plant height by 86.2 or 65.0%, total chlorophyll by 73 or 50%, proline by 35 or 45%, glutathione by 100 or 138%, SY by 625 or 463% and EOY by 300 or 335%, respectively, in fennel plants. Co-application of the naturally occurring microorganisms (i.e., LAB) and organically-derived, nutrient-rich fertilizer (i.e., FM or PM) is recommended to improve yield of fennel plants in saline calcareous soils.

## Introduction

Medicinal plants, especially those that can serve as natural antioxidants, have recently attracted attention due to their increasing health and safety concerns^1,2^. Fennel (*Foeniculum vulgare* Mill.) is a winter crop native to the Mediterranean region that belongs to *Apiaceae* family^3^. It is cultivated as an aromatic or vegetable crop in arid and semi-arid regions^4^. Globally, the total harvested area of fennel in 2020 was 1.96 Mha, yielding about 2.22 MT of seeds^5^. In Egypt, fennel is considered a promising “unconventional” vegetable crop for exportation and local consumption^6^. This aromatic crop covers 32,534 ha yielding 28,923 tonnes of seeds, averaging 0.89 tonnes ha^−1^ in the winter season of 2020^5^.

It is widely known that inflorescences, leaves, and stems of fennel plants contain substances that have antibacterial, hepatic and antioxidant properties^3^. In general, fennel is rich in potassium (K), calcium (Ca) and vitamins A, B and C which are essential for metabolism^4^. Besides, it can be used in food industry, beverages, folk medicine, animal fodder, cosmetic products and perfumery^6–8^. Due to the aromatic chemicals (anethole, estragole and fenshon) present in its essential oil (EO), fennel has many pharmacological effects, including antioxidant, anticancer, anti-inflammatory, antifungal, antibacterial and estrogenic activities^9^. 

Salinity is a significant abiotic stress that affects crop productivity^10–13^. In arid and semi-arid areas, soil salinity reduces soil fertility and water uptake in crop plants, leading to limited agricultural production^14–17^. It has been reported that over 20% of the irrigated land will be off-duty, and about 50% of the cultivable land will be affected by salinity by 2050^18^.

Saline calcareous soil covers a considerable portion of agricultural desert land, including Egypt. It has an electrical conductivity of saturated soil extract (ECe) of > 4 dS m^−1^^19^. It also contains excess concentrations of calcium carbonate (CaCO_3_) and magnesium carbonate (MgCO_3_) of 14–17% and tends to be low in organic matter (OM) content and available nitrogen (N). The high alkalinity of soil may result in low availability and solubility of macroelements and micronutrient, which in turn limits crop productivity^20^. Due to salinity and lack of soil nutrient resources, especially in calcareous soil, cultivation of fennel crop and seed production have been seriously affected^21^.

Biostimulants can help plants lessen the impact of abiotic stress^22^. Organic amendments applied seasonally are the best agronomic strategy to improve the biological, chemical and physical characteristics of soils, and enhance crop tolerance and productivity in saline calcareous soils^10,23^.

In addition, biological (microbial) treatments can be used as a low-cost and eco-friendly method for food security and agricultural sustainability^24^. Plant microbiome (also known as phytomicrobiome) engineering is an alternative approach that has positive effect on plant growth, development and productivity under extreme conditions^24–26^. Lactic acid bacteria (LAB) are known for their antagonistic and plant growth-promotion activities^27–29^. They are involved in improving soil structure, OM contents and nutrient uptake; thus, reducing the need of chemical fertilizers/pesticides^10^. There is evidence that certain LAB strains, mainly *Lactobacillus*, can help plants adapt to abiotic stress.

We hypothesize that the combined application of organic manure [farmyard manure (FM) or poultry manure (PM)] and LAB [*Lactobacillus plantarum (Lp*) and *Lactococcus lactis (Ll*)] could improve not only soil fertility, but also crop productivity of salinity stressed-fennel plants. The purpose of this study was to determine the role of FM or PM as soil amendments solely or in combination with *Lp* and/or *Ll* on the properties of soil, and growth, chemical constituents and essential oil yield (EOY) of fennel plants grown in saline calcareous soil. This research will reduce the use of chemical fertilizers and relieve plants from environmental pollution to promote sustainable development of agriculture.

## Materials and methods

### Site of experiment

Open field trials were carried out on October 27–May 8 of 2019/2020 and 2020/2021 in the Research Station, Fayoum Governorate, Egypt, at latitudes (29° 17′N), longitudes (30° 53′E). During the experimental period, the environmental characteristics were as the following: Average temperature = 25 ± 3 °C day/10 ± 2 °C night, relative humidity = 45 ± 4%, and average daylight length = 11 h. The soil used in the experimental site was sandy loam in texture (77.53% sand, 10.10% silt and 12.37% clay), bulk density (BD) = 1.55 g cm^−3^ and retained available water (AW) = 12.13%.

The soil was saline (ECe = 8.53 dS m^−1^)^30,31^, CaCO_3_ = 14.86%, exchangeable sodium percentage (ESP) = 15.62 and pH = 7.69. The OM content and total N in the soil were 1.12% and 0.03%, respectively. All physiochemical analyses of the studied soil were carried out according to previously used methods^32,33^. The total count of bacteria was performed as previously described^34^.

### Plant material and organic manure fertilizers

Fennel seeds were obtained from the Institute of Medicinal and Aromatic Plants, Agricultural Research Center (ARC), Giza, Egypt. The use of plants or plant parts, in the present study, complies with the international, national and/or institutional guidelines. Organic manure fertilizers (FM and PM) were purchased from private farms for cattle and poultry production that are based in Fayoum city, Egypt.

According to the commercial agronomic practices of fennel in this region, 25 and 20 t ha^−1^ of FM and PM were used, respectively. Chemical properties of the used FM and PM were summarized in Table S1.

### LAB strains

*Lactobacillus plantarum* subsp*. plantarum* ATCC 14917 (*Lp)* and *Lactococcus lactis* subsp*. lactis* ATCC 11454 (*Ll*) were obtained from Agricultural Microbiology Department, Faculty of Agriculture, Fayoum University, Fayoum, Egypt. Both strains were cultivated on de Man, Rogosa and Sharpe (MRS) agar (Lab M Limited, Lancashire, UK) and stored at 4 °C. In order to obtain cell suspensions of bacterial strains, each strain was inoculated into double-strength MRS broth, and then the mixture was incubated at 37 °C for one night. The final concentration of cells reached 5 × 10^9^ colony forming units (CFU) mL^−1^.

The characteristics of the two *Lactobacillus* strains are presented in Table S2. Fennel seeds were then inoculated by adding 100 mL of cell suspensions of each *Lactobacillus* strain to a 250 mL flask and the flasks were incubated at 37 °C for 24 h. For the treatment of the combined LAB strains, fennel seeds were inoculated with 1:1 ratio with the mixture of cell suspension of *Lp* and *Ll*.

### Experimental setup

In the in vivo experiment, the effect of organic manure amendments and LAB on fennel plants cultivated under saline calcareous soil was evaluated. In addition to the control (no organic manure or LAB), the treatments included two organic manures (FM and PM) applied (as recommended above) individually or combined with one or both *Lp* and/or *Ll*, as mentioned above. In addition, to the control treatment, the individual/combinations of treatments were applied, as presented in Table 1.

**Table 1:** Treatments applied in the current study during the winter seasons of 2019/2020 and 2020/2021.

Treatment	Abbreviation
Control	C
Farmyard manure	FM
Farmyard manure + *Lactobacillus plantarum*	FM + *Lp*
Farmyard manure + *Lactococcus lactis*	FM + *Ll*
Farmyard manure + *L. plantarum* + *L. lactis*	FM + *Lp* + *Ll*
Poultry manure	PM
Poultry manure + *L. plantarum*	PM + *Lp*
Poultry manure + *L. lactis*	PM + *Ll*
Poultry manure + *L. plantarum* + *L. lactis*	PM + *Lp* + *Ll*

Two field trials were set in a randomized complete block design (RCBD). Treatments were replicated four times, making a total of 36 plots. The area of the experimental plot was 9 m^2^ (3 × 3 m row). Each plot contained five lines (3 m in length and 0.6 m apart). On the 27th of October of both seasons, inoculated fennel seeds were sowed in hills of 30 cm apart at a rate of 3–5 seeds hill^−1^. At 21 days after sowing (DAS), each hill was thinned to have 2–3 seedlings, and repeated later on 45 DAS to have vigorous seedlings hill^−1^. Fennel plants were harvested manually on the 8th of May of both seasons.

The recommended doses of fertilizers were applied, as the following: Phosphorus fertilizer = 75 kg P_2_O_5_ ha^−1^ was applied during seedbed preparation, N fertilizer = 150 kg N ha^−1^ was applied in two equal doses on 45 and 75 DAS, and K fertilization = 50 kg K_2_O ha^−1^ on 75 DAS. Foliar application of chelated micronutrient solution fertilizer (Disper Complex GS, Sphinx International Trade Co., Nasr city, Egypt) = 0.5 g L^−1^ was sprayed twice on fennel seedlings on 40 and 70 DAS.

### Agronomic parameters of fennel plants

The vegetative growth characteristics were estimated at the full blooming stage (90 DAS). Five plants were randomly chosen from each plot to measure plant height (PH; cm) and root length (RL; cm), and the number of main branches plant^−1^ were also counted. Fresh weight (FW; g plant^−1^) of shoots and roots was recorded using a digital scale, while dry weight (DW; g plant^−1^) was noted after oven-drying at 70 °C for constant weight.

At the end of the experiment (on May 8), five plants were dedicated to assess yield traits of the number of umbels plant^−1^, biological yield (BY; t ha^−1^), seed yield (SY; t ha^−1^) and seed index (SI; weight of 1000 seeds; g). The following equation was used to determine the harvest index (HI %).

$$\mathrm{HI} (\%)=\frac{\mathrm{SY}}{\mathrm{BY}} \times 100$$

### Biochemical analyses

At 90 DAS, leaf pigments, including chlorophyll (Chl) *a* and *b*, and total carotenoids (Car) were measured (mg g^−1^ FW)^35^. Shoot DW was used to colorimetrically determine the free proline (Pro) content (mg g^−1^ DW)^36^ and total soluble sugars (TSS; mg g^−1^ DW) was estimated using phosphomolybdic acid reagent^37^. Total phenolic content was measured by the Folin-Ciocalteu colorimetric technique using the methanolic extract of the identical dry material^38^. Kjeldahl digestion (Ningbo Medical Instruments Co., Ningbo, China) was used to assess N concentration. The amount of P was quantified using the molybdenum-reduced molybdophosphoric blue color technique^39^.

A Perkin-Elmer Model 52-A flame photometer (PerkinElmer, Inc., Waltham, Massachusetts, USA) was used to measure the amounts of K^+^ and Na^+^ in samples^40^. Perkin-Elmer atomic absorption spectrophotometer was used to determine Ca^2+^ concentration^41^. Phenolic components were extracted from dried tissues according to the procedure of Sauvesty et al.^42^. The Folin–Ciocalteau phenol method^43^ was used to determine the phenolic aglycone. Soluble proteins were also extracted and measured^44,45^. To estimate lipid peroxidation of fennel leaves, malondialdehyde (MDA) content was measured by the thiobarbituric acid assay as previously defined^46^.

Total flavonoids content (TFC) was measured colorimetrically using the method of Lamaison and Carnet^47^, and 2,2-diphenyl-1-picrylhydrazyl (DPPH) radical-scavenging activity (DPPH RSA) of the extract was calculated by DPPH free radical^48^.

### EOY

To determine EOY, 250 g of air-dried fruits from each plot were crushed in a grinder with 500 mL distilled water, and the yield (mL 100 g^−1^) was estimated by water distillation in a Neo-Clevenger type apparatus^49^. Following that, EOY (%)/weight was calculated.

### Statistical analysis

Data collected were statistically examined following the analysis of variance (ANOVA) procedure^50^ using Microcomputer Statistical Package (MSTAT-C; East Lansing, Michigan, USA). Mean values were compared using Duncan's multiple range test at a 0.05 probability level (*P* ≤ 0.05) to determine the statistical significance.

## Results

### Soil properties and total bacterial count

Soil properties and total bacterial counts were significantly (*P* ≤ 0.05) affected when the soil was amended with organic manures and LAB strains (Table 2). Applying FM or PM individually or combined with either *Lp* or *Ll* significantly decreased ECe, BD and soil pH values. However, the same treatments increased OM, N and P contents, total porosity (TP), soil water contents at field capacity (θ_Fc_) and AW.

**Table 2:** Effect of the application organic manures and LAB on soil physicochemical properties after harvesting stage (averaged over the two seasons of 2019/2020 and 2020/2021).

Treatments	Total bacterial count log_10_ (CFU g^−1^)	pH	ECe (dS m^−1^)	OM (%)	N	P	BD (g cm^−3^)	TP	θ_Fc_	AW
					(mg kg^−1^ )			(%)		
C	5.82 ± 0.04d	7.86 ± 0.11a	6.92 ± 0.11a	1.16 ± 0.06e	51.0 ± 1.50e	3.53 ± 0.32e	1.61 ± 0.12a	32.1 ± 1.2d	16.8 ± 1.10d	9.71 ± 0.08d
FM	6.14 ± 0.35c	7.60 ± 0.12c	5.81 ± 0.12b	1.63 ± 0.07c	59.2 ± 2.10d	4.26 ± 0.19e	1.56 ± 0.11a	36.2 ± 1.60c	23.2 ± 1.20b	13.42 ± 0.09c
FM + *Lp*	6.29 ± 0.07b	7.51 ± 0.21d	5.70 ± 0.08c	1.84 ± 0.07b	61.4 ± 2.10c	4.64 ± 0.21c	1.55 ± 0.09b	36.7 ± 1.60c	24.0 ± 1.70a	14.31 ± 0.11 a
FM + *Ll*	6.14 ± 0.35c	7.52 ± 0.14d	5.60 ± 0.10c	1.81 ± 0.06b	62.3 ± 2.40c	4.55 ± 0.22c	1.57 ± 012a	36.8 ± 1.20c	24.1 ± 1.80a	14.22 ± 0.10a
FM + *Lp* + *Ll*	7.46 ± 0.40a	7.44 ± 0.16d	5.61 ± 0.16c	2.01 ± 0.10a	67.2 ± 1.40a	5.28 ± 0.31a	1.5 ± 0.14c	39.2 ± 1.70a	24.3 ± 1.40a	14.45 ± 0.13a
PM	6.12 ± 0.50c	7.70 ± 0.23b	5.91 ± 0.17b	1.56 ± 0.11d	61.3 ± 2.00c	4.39 ± 0.18d	1.54 ± 0.15b	36.7 ± 1.60c	22.3 ± 1.30c	13.62 ± 0.11c
PM + *Lp*	6.33 ± 0.28b	7.61 ± 0.22c	5.72 ± 0.13c	1.68 ± 0.09c	64.2 ± 1.80b	4.43 ± 0.21d	1.53 ± 0.16b	36.8 ± 1.40c	23.6 ± 1.20b	14.10 ± 0.12a
PM + *Ll*	6.10 ± 0.20c	7.60 ± 0.18c	5.84 ± 0.14b	1.87 ± 0.08b	63.5 ± 1.70b	4.60 ± 0.24c	1.54 ± 0.14b	36.9 ± 1.30c	24.1 ± 1.30a	13.91 ± 0.11b
PM + *Lp* + *Ll*	7.25 ± 0.25a	7.51 ± 0.17d	5.73 ± 0.13c	1.90 ± 0.11a	66.2 ± 1.60a	4.81 ± 0.28b	1.49 ± 0.12c	37.1 ± 1.70a	24.0 ± 1.60a	14.11 ± 0.13a

All abbreviations for treatments can be found in Table 1.

Mean values (± SE) with different letters within a column are significantly different at *P* ≤ 0.05 according to Duncan’s multiple range test.

LAB, lactic acid bacteria; CFU, colony forming unit; ECe, electrical conductivity of soil extract; OM, organic matter; N, nitrogen; P, phosphorus; BD, bulk density; TP, total porosity; θ_Fc_, field capacity; AW, available water.

The addition of FM + *Lp* + *Ll* further increased the abovementioned soil properties by 73.3, 31.4, 49.6, 22.81, 42.9, and 48.8%, respectively, compared to the control (Table 2). PM + *Lp* + *Ll* also increased OM, N and P ontents, TP, θ_Fc,_ and AW by 36.3, 63.8, 29.8, 15.6, 42.9, and 45.3%, respectively, compared to plants not treated with any organic manure or LAB (control). Total bacterial counts improved in soils treated with FM + *Lp* + *Ll* or PM + *Lp* + *Ll*; of which this improvement was clearly obvious in plants treated with FM + *Lp* + *Ll* with an increase that reaching to 28.2% compared to the control treatment (Table 2).

### Characteristics of vegetative growth

In saline calcareous soils, PH, number of main branches, RL, shoot and root FW and shoot and root DW of fennel plants were significantly (*P* ≤ 0.05) enhanced by 18.3 or 21.3%, 42.4 or 24.2%, 18.4 or 11.8%, 119.5 or 123.0%, 43.0 or 67.7%, 78.0 or 58.0%, and 45.5 or 23.3% when the soil was amended with the recommended dose of FM or PM, compared to non-amended plants (Table 3).

**Table 3:** Effect of organic manure amendments and LAB on the vegetative growth characteristics of fennel plants (*Foeniculum vulgare* Mill.) grown in saline calcareous soil.

Treatment	PH (cm)	Number of branches	RL (cm)	Shoot (g plant^−1^)		Root (g plant^−1^)	
				FW	DW	FW	DW
C	98.7 ± 2.5f	3.3 ± 0.2g	13.6 ± 0.6g	236.3 ± 1.5g	128.3 ± 1.5h	50.0 + 1.7h	33.0 ± 2.9g
FM	116.8 ± 2.9e	4.7 ± 0.2ef	16.1 ± 0.3f	299.2 ± 2.1f	162.0 ± 1.7g	89.0 + 2.1f	48.0 ± 2.1e
FM + *Lp*	139.8 ± 3.1cd	6.6 ± 0.4c	18.7 ± 0.9e	409.7 ± 3.9d	209.0 ± 2.3e	119.7 + 2.9e	60.3 ± 2.0d
FM + *Ll*	144.8 ± 3.2c	5.3 ± 0.2de	23.5 ± 0.5c	373.3 ± 4.5e	184.0 ± 2.6f	118.3 + 2.6e	48.7 ± 2.3e
FM + *Lp* + *Ll*	183.8 ± 2.9a	9.8 ± 0.3a	29.8 ± 0.4a	844.7 ± 7.3a	466.7 ± 3.5a	229.7 + 2.8a	98.7 ± 2.9a
PM	119.7 ± 3.3e	4.1 ± 0.3fg	15.2 ± 0.3fg	304.0 ± 3.5f	181.3 ± 2.0f	79.0 + 2.5g	40.7 ± 2.6f
PM + *Lp*	132.8 ± 2.9d	6.7 ± 0.2c	20.7 ± 0.6d	409.0 ± 4.2d	229.0 ± 2.6d	141.7 + 2.4c	73.0 ± 2.9c
PM + *Ll*	133.6 ± 2.1d	5.7 ± 0.2d	22.6 ± 0.6c	549.0 ± 5.8c	295.7 ± 2.3c	127.0 + 2.9d	71.5 ± 2.4c
PM + *Lp* + *Ll*	162.6 ± 3.0b	8.1 ± 0.4b	27.9 ± 0.5b	794.0 ± 6.4b	399.0 ± 3.1b	159.0 + 3.2b	84.5 ± 2.9b

All abbreviations for treatments can be found in Table 1.

Mean values (± SE) with different letters within a column are significantly different at *P* ≤ 0.05 according to Duncan’s multiple range test.

LAB, lactic acid bacteria; PH, plant height; RL, root length; FW, fresh weight; DW, dry weight.

Furthermore, inoculated fennel seeds with *Lp*, *Ll* or *Lp* + *Ll* mixture markedly (*P* ≤ 0.05) increased the abovementioned growth under in soils amended with FM or PM, compared to control (Table 3). Strikingly, the triple combinations of FM + *Lp* + *Ll* and PM + *Lp* + *Ll* caused the most significant (*P* ≤ 0.05) increases in all these parameters: PH by 86.2 and 64.7%, number of main branches by 197.0 and 145.5%, RL by 119.1 and 105.1%, shoot FW by 257.5 and 236.0%, shoot DW by 263.8 and 211.0%, root FW by 229.7 and 159.0%, and root DW by 199.1 and 156.1%, respectively, compared to control plants (Table 3).

### Biochemical constituents of fennel

When FM or PM supplemented into the saline calcareous soil, this resulted in significant (*P* ≤ 0.05) increases in the total contents of Chl, Car, TSS, free amino acids (FAA) and total proteins (TPs) of fennel plants by 11.5 or 26.9%, 50.0 or 68.8%, 11.7 or 15.3%, 10.0 or 20.0%, and 54.2 or 60.4%, respectively, compared to non-amended plants (Table 4). However, Pro content decreased by 33.7 or 28.5% in plants treated with FM or PM, respectively. In response to the mixture of organic amendments (with either or both tested LAB strains, a remarkable increase in leaf pigment characteristics was observed compared with that in control plants (Table 4).

**Table 4:** Effect of organic manure amendments and LAB on photosynthetic efficiency, osmoprotectants and soluble protein content of fennel plants (*Foeniculum vulgare* Mill.) grown in saline calcareous soil.

Treatment	Total Chl	Total Car	TSS	FAA	TPs	Pro
	(mg g^−1^ FW)		(mg g^−1^ DW)			
C	2.6 ± 0.10f	0.32 ± 0.01f	0.96 ± 0.01f	0.10 ± 0.00g	0.48 ± 0.02g	1.93 ± 0.04a
FM	2.9 ± 0.10e	0.48 ± 0.01e	1.06 ± 0.02e	0.11 ± 0.00f	0.74 ± 0.03f	1.28 ± 0.03ef
FM + *Lp*	3.4 ± 0.10cd	0.65 ± 0.01cd	1.11 ± 0.03de	0.12 ± 0.00de	0.82 ± 0.02e	1.61 ± 0.06b
FM + *Ll*	3.7 ± 0.10bc	0.74 ± 0.01bc	1.17 ± 0.02cd	0.13 ± 0.00c	1.09 ± 0.03c	1.46 ± 0.04cd
FM + *Lp* + *Ll*	4.5 ± 0.10a	1.08 ± 0.01a	1.30 ± 0.03b	0.18 ± 0.00a	1.78 ± 0.03a	1.24 ± 0.02f
PM	3.3 ± 0.10d	0.54 ± 0.01de	1.07 ± 0.03e	0.12 ± 0.00d	0.77 ± 0.02ef	1.38 ± 0.04de
PM + *Lp*	2.9 ± 0.10e	0.63 ± 0.01cde	1.19 ± 0.02c	0.11 ± 0.00f	0.98 ± 0.02d	1.50 ± 0.01c
PM + *Ll*	3.3 ± 0.10d	0.77 ± 0.01bc	1.22 ± 0.03c	0.12 ± 0.00ef	1.08 ± 0.01c	1.39 ± 0.03d
PM + *Lp* + *Ll*	3.9 ± 0.10b	0.90 ± 0.01b	1.39 ± 0.02a	0.15 ± 0.00b	1.24 ± 0.02b	1.11 ± 0.03g

All abbreviations for treatments can be found in Table 1.

Mean values (± SE) with different letters within a column are significantly different at *P* ≤ 0.05 according to Duncan’s multiple range test.

LAB, lactic acid bacteria; Chl, chlorophyll; Car, carotenoids; TSS, total soluble sugars; FAA, free amino acids; TPs, total protein contents; Pro, proline; FW, fresh weight; DW, dry weight.

Our results showed that the mixture FM + *Lp* + *Ll* or PM + *Lp* + *Ll* was pronounced the highest values of total Chl (73.1 or 50.0%) and Car (237.5 or 181.3%), respectively, compared to the control (Table 4). Similarly, fennel seeds treated with a mixture of *Lp* + *Ll* strains produced significantly (*P* ≤ 0.05) higher TSS, FAA and TPs, and lower Pro levels in dry tissues of fennel seedlings when soils were amended with FM or PM than non-amended ones (Table 4).

### Non-enzymatic antioxidant activity

Fennel plants previously inoculated with *Lp* + *Ll* in combination with FM or PM recorded the highest level of TFC, while plants treated with FM without any seed inoculation showed the lowest level of TFC of all treatments in comparison to untreated plants (Table 5). Plants treated with PM + *Lp *+ *Ll *and FM + *Lp *+ *Ll *had the highest DPPH RSA values when compared to salt-stressed fennel plants without any amendments (Table 5).

**Table 5:** Effect of organic manure amendments and LAB on TFC, DPPH RSA, phenolics and non-enzymatic antioxidant activity of fennel plants (*Foeniculum vulgare* Mill.) grown in saline calcareous soil.

Treatment	TFC (µg Rutin equivalent /g^−1^ DW)	DPPH RSA (%)	Phenolics		Antioxidants		MDA (µM mL^−1^)
			Glycon (mg GAE/g^−1^ DW)	Aglycon (mg GAE/g^−1^ DW)	AsA (µM mL^−1^)	GSH (µM mL^−1^)	
C	7.18 ± 0.01i	79.98 ± 0.02h	0.14 ± 0.00h	0.22 ± 0.00a	0.14 ± 0.00f	0.08 ± 0.01f	0.11 ± 0.00a
FM	9.47 ± 0.03h	82.49 ± 0.07g	0.17 ± 0.00g	0.18 ± 0.00c	0.15 ± 0.00e	0.10 ± 0.01e	0.08 ± 0.00b
FM + *Lp*	10.66 ± 0.05f	84.52 ± 0.09e	0.25 ± 0.00c	0.16 ± 0.00e	0.15 ± 0.00e	0.14 ± 0.01cd	0.06 ± 0.00c
FM + *Ll*	11.44 ± 0.05e	84.52 ± 0.10e	0.21 ± 0.00e	0.17 ± 0.00d	0.17 ± 0.00c	0.15 ± 0.01bc	0.04 ± 0.00d
FM + *Lp* + *Ll*	16.37 ± 0.06b	93.23 ± 0.10b	0.27 ± 0.00b	0.14 ± 0.00g	0.18 ± 0.00b	0.16 ± 0.01b	0.01 ± 0.00f
PM	9.88 ± 0.04g	84.15 ± 0.08f	0.18 ± 0.00f	0.20 ± 0.00b	0.15 ± 0.00ef	0.12 ± 0.01d	0.07 ± 0.00b
PM + *Lp*	13.07 ± 0.06d	86.52 ± 0.11d	0.25 ± 0.00c	0.18 ± 0.00c	0.16 ± 0.00d	0.12 ± 0.01d	0.06 ± 0.00c
PM + *Ll*	13.73 ± 0.06c	87.01 ± 0.10c	0.23 ± 0.00d	0.17 ± 0.00d	0.17 ± 0.00c	0.15 ± 0.01bc	0.03 ± 0.00e
PM + *Lp* + *Ll*	18.18 ± 0.05a	95.33 ± 0.12a	0.30 ± 0.00a	0.15 ± 0.00f	0.19 ± 0.00a	0.19 ± 0.01a	0.02 ± 0.00f

All abbreviations for treatments can be found in Table 1.

Mean values (± SE) with different letters within a column are significantly different at *P* ≤ 0.05 according to Duncan’s multiple range test.

LAB, lactic acid bacteria; TFC, total flavonoids content; DPPH, 2,2-diphenyl-1-picrylhydrazyl; RSA, radical-scavenging activity; AsA, ascorbate; GSH, glutathione; MDA, malondialdehyde; DW, dry weight; GAE = gallic acid equivalent.

Fennel plants cultivated in saline calcareous soil and amended with either FM or PM considerably (*P* ≤ 0.05) improved the accumulation of non-enzymatic phenolic compound (glycon), and the antioxidants [ascorbate (AsA) and glutathione (GSH)] in fennel tissues by 21.4 or 28.6%, 7.1 or 7.1% and 25.0 or 50.0%, respectively, compared to control plants (Table 5). On the other hand, there was a decrease in the phenolic-aglycon by 18.2 or 9.1% in plants amended with FM or PM, respectively, related to un-amended plants. By providing *Lp* or *Ll* individually or in mixture markedly (*P* ≤ 0.05) increased the accumulation of non-enzymatic antioxidant compounds in fennel dry tissues, compared to the control (Table 5). The combined treatment of FM + *Lp* + *Ll* or PM + *Lp* + *Ll* on fennel significantly (*P* ≤ 0.05) increased phenolic-glycon by 92.9 or 114.3%, AsA by 28.6 or 35.7%, and GSH by 100.0 or 137.5%, respectively, compared untreated control plants. On the other hand, the same treatments decreased phenolic-aglycon by 36.4 or 31.8%, respectively (Table 5).

Our results showed that any of the organic manures either applied alone or mixed with individual LAB strain significantly decreased MDA content in plants cultivated in saline calcareous soil (Table 5). For example, plants treated with FM + *Lp* + *Ll* or PM + *Lp* + *Ll* displayed the minimum lipid peroxidation activity (i.e., MDA) by 0.01 or 0.02 µM mL^−1^, respectively; whereas the highest MDA content was obtained in the control treatment (0.11 µM mL^−1^ of MDA) (Table 5).

### SY and its components

In general, the amendment of FM or PM to saline calcareous soil enhanced SY in fennel plants. This is evident when the number of umbels plant^−1^ was increased by 44.1 or 39.4%, BY by 28.6 or 38.1%, SY by 87.5 or 112.5%, HI by 47.8 or 59.2%, SI by 31.4 or 60.0%, and EOY by 187.0 or 121.7%, in plants treated with either FM or PM, respectively, compared to control (Table 6).

**Table 6:** Effect of organic manure amendments and LAB on yield components of fennel plants (*Foeniculum vulgare* Mill.) grown in saline calcareous soil.

Treatment	Number of umbels plants^−1^	BY (t h^–1^)	SY (t h^–1^)	HI (%)	SI (g)	EOY (%)
C	12.7 ± 0.30f	4.2 ± 0.01i	0.8 ± 0.05g	18.4 ± 0.50g	3.5 ± 0.03h	0.46 ± 0.01h
FM	18.3 ± 0.70e	5.4 ± 0.04h	1.5 ± 0.07f	27.2 ± 0.60f	4.6 ± 0.04g	1.32 ± 0.01f
FM + *Lp*	24.3 ± 0.70d	8.6 ± 0.04c	2.6 ± 0.06d	30.3 ± 0.70e	5.8 ± 0.03d	1.52 ± 0.01e
FM + *Ll*	28.3 ± 0.90c	7.9 ± 0.06d	3.1 ± 0.07c	38.8 ± 0.50d	6.2 ± 0.02c	1.58 ± 0.01d
FM + *Lp* + *Ll*	35.7 ± 1.2a	11.5 ± 0.09a	5.8 ± 0.03a	50.4 ± 0.60a	7.6 ± 0.03a	1.84 ± 0.01b
PM	17.7 ± 0.90e	5.8 ± 0.05g	1.7 ± 0.06e	29.3 ± 0.50e	5.6 ± 0.02e	1.02 ± 0.01g
PM + *Lp*	22.7 ± 0.90d	6.4 ± 0.05f	2.6 ± 0.07d	39.8 ± 0.80c	4.6 ± 0.04g	1.52 ± 0.01e
PM + *Ll*	25.3 ± 0.90d	7.7 ± 0.05e	3.0 ± 0.04c	38.6 ± 0.40d	5.2 ± 0.04f	1.64 ± 0.01c
PM + *Lp* + *Ll*	32.3 ± 1.50b	9.5 ± 0.08b	4.5 ± 0.03b	47.7 ± 0.50b	6.3 ± 0.04b	2.00 ± 0.01a

All abbreviations for treatments can be found in Table 1.

Mean values (± SE) with different letters within a column are significantly different at *P* ≤ 0.05 according to Duncan’s multiple range test.

LAB, lactic acid bacteria; BY, biological yield; SY, seed yield; HI, harvest index; SI, seed index (weight of 1000 seeds); EOY, essential oil yield.

Fennel plants treated with FM + *Lp*, FM + *Ll*, FM + *Lp* + *Ll*, PM + *Lp*, PM + *Ll* or PM + *Lp* + *Ll* further augmented the above-mentioned attributes in comparison with control plants. This was evident when PM + *Lp* + *Ll* treatment considerably (*P* ≤ 0.05) increased BY and SY, HI and SI by 126.2, 462.5, 159.2 and 80.0%, respectively, compared to un-amended fennel plants (Table 6). The same traits also increased by 173.8% (BY), 625.0% (SY), 173.9% (HI), and 117.1% (SI), respectively, when amended with FM + *Lp* + *Ll* (Table 6). For EOY, the highest increment was 334.8% recorded in fennel plants treated with PM + *Lp* + *Ll*, followed by 300.0% in the treatment obtained from FM + *Lp* + *Ll*.

### Leaf mineral contents

Nutrient contents in the dry tissues were also measured to assess nutrient uptake in fennel plants. Compared to other soil amendments and/or LAB treatments , saline-stressed fennel plants (control) exhibited low levels in N, P, K^+^ and Ca^2+^ contents and K^+^/Na^+^ ratio, but huge accumulation of Na^+^ (Table 7). On the other hand, application of FM or PM to the soil markedly (*P* ≤ 0.05) increased the uptake of N by 13.6 or 63.5%, P by 57.6 or 83.1%, K^+^ by 41.9 or 43.4%, Ca^2+^ by 20.4 or 8.8%, and leaf K^+^/Na^+^ ratio by 78.9 or 81.6%, but decreased leaf Na^+^ content by 20.2 or 20.0%, respectively, compared to control plants (Table 7).

**Table 7:** Effect of organic manure amendments and LAB and on leaf macro mineral content of fennel plants (*Foeniculum vulgare* Mill.) grown insaline calcareous soil.

Treatment	N	P	K^+^	Na^+^	Ca^2+^	K^+^/Na^+^
	(%)	(mg g DW^–^^1^)				
C	2.2 ± 0.03h	1.42 ± 0.03g	13.97 ± 0.16f	36.30 ± 0.22a	9.87 ± 0.17g	0.38 ± 0.01f
FM	2.5 ± 0.04g	2.24 ± 0.04f	19.83 ± 0.36c	28.97 ± 0.25b	11.88 ± 0.21e	0.68 ± 0.01c
FM + *Lp*	3.4 ± 0.03e	3.00 ± 0.05d	18.47 ± 0.45d	26.85 ± 0.20e	13.90 ± 0.26c	0.69 ± 0.02c
FM + *Ll*	3.5 ± 0.04de	3.57 ± 0.03c	17.92 ± 0.25d	27.98 ± 0.23cd	12.63 ± 0.25de	0.64 ± 0.01cd
FM + *Lp* + *Ll*	4.8 ± 0.06a	5.09 ± 0.05a	24.52 ± 0.65a	23.65 ± 0.21g	16.45 + 0.32a	1.04 ± 0.02a
PM	3.6 ± 0.05d	2.60 ± 0.04e	20.03 ± 0.45c	29.03 ± 0.25b	10.74 ± 0.25f	0.69 ± 0.01c
PM + *Lp*	3.2 ± 0.05f	2.47 ± 0.04e	15.90 ± 0.25e	28.62 ± 0.25bc	13.41 ± 0.25cd	0.56 ± 0.01e
PM + *Ll*	3.8 ± 0.04c	3.11 ± 0.07d	17.38 ± 0.47d	27.87 ± 0.20d	12.69 ± 0.22d	0.62 ± 0.02d
PM + *Lp* + *Ll*	4.0 ± 0.05b	4.17 ± 0.05b	21.90 ± 0.29b	25.70 ± 0.18f	15.55 ± 0.23b	0.85 ± 0.03b

All abbreviations for treatments can be found in Table 1.

Mean values (± SE) with different letters within a column are significantly different at *P* ≤ 0.05 according to Duncan’s multiple range test.

LAB, lactic acid bacteria; N, nitrogen; P, phosphorus; K, potassium; Na, sodium; Ca, calcium; DW, dry weight.

In addition, fennel seeds previously inoculated with *Lp* + *Ll* and organically amended with FM or PM had the maximum leaf N, P, K^+^and Ca^2+^ contents, and K^+^/Na^+^ ratio by 118.2 or 81.8%, 258.5 or 193.7%, 75.5 or 56.8%, 66.7 or 57.5% and 173.7 or 123.7%, respectively, compared to non-treated plants (Table 7). The minimum content of Na^+^ in fennel leaves was meaured in fennel plants teated with FM + *Lp* + *Ll* (by 34.8%) or PM + *Lp* + *Ll* (by 29.2%) compared to control plants sown in saline calcareous soil (Table 7).

## Discussion

Climate change and poor soil salinity management affect agricultural productivity and ecological balance; thus, undermining sustainability and food security. Effective microorganisms and organic amendments are very helpful to alleviate the harmful effect of soil salinity. Organic additives provide macro- and micronutrients, increase water holding capacity and cation exchange capacity, and change pH of soils; in addition to increasing microbial activities and nutrient recycle^11^. In the current study, applying FM or PM to saline calcareous soil individually or in combination with seed inoculation with LAB revealed effective physio-chemical properties of the tested soil, and better growth and productivity of fennel plants. The application of FM or PM to saline calcareous soil not only decreased ECe, but also increased overall bacterial counts. These results are consistent with those of^51^ who have reported that improving soil physical features may enable soil particles to associate with each other in an aggregated manner, which may enhance overall soil health. The mixture of any organic manure with any of LAB strains -used in the present study- led to drop in BD, and increase in TP and salt leaching; leading to mitigation of high salt stress in fennel plants.

Due to the release of organic acids and carbon dioxide from the decomposing FM or PM, soil pH decreased. Similar to the results obtained by other reports^52^, there was an increase in OM, N and P, when fennel plants were treated with PM + *Lp* + *Ll*. Previously, Zhang et al.^53^ have reported that applying FM or PM combined with *Lp* or *Ll* to the soil can improve microbial activity and plant growth. Our results showed that agronomical growth parameters of fennel plants, including PH, number of main branches, FW and DW of shoots and roots, significantly increased when organic manures were mixed with LAB and supplemented to saline calcareous soil. Plant growth can be attributed to the organic manure applied, which affects plant growth and plant growth regulators, including hormones, vitamins and amino acids^54^. Due to the availability and readily uptake of nutrients by plants, organic manures can improve plant growth and development^55^. The medicinal plant *Swertia chirayita* inoculated with *Lp* (ATCC 9019) showed altered metabolic responses and increased tolerance to salt stress^56^. The ability of LAB strains to produce polyamines may also contribute to growth promotion and stress alleviation in plants^57^. In addition, LAB can produce cytokinins, auxins and other hormones to stimulate plant growth^58^.

Inhibition of fennel growth is usually associated with increased salinity and CaCO_3_ in soils, which can be attributed to the decreased Chl content, reactive oxygen species (ROS)-induced chlorosis and photo-reduction, causing serious damage to photosystems I and II and formation of Chl in plants^59^. FM or PM incorporated with LAB enhanced the photosynthetic capacity of total Chl or Car of fennel plants grown in saline calcareous soils. In another study carried out in saline soils, the photosynthesis efficiency of sweet sorghum (*Sorghum bicolor*) increased with organic manure supplementation^18^. Photochemical efficiency and total Chl content (SPAD) significantly increased with soil organic amendments under salt stress conditions^10^. This could be due to the synergistic effect of the consortia, which can improve plant N, P and K uptake, resulting in increased Chl accumulation^60,61^.

Fennel plants cultivated in saline calcareous soil induced positive effects on osmotic potential of plants when supplemented with LAB; thus, increasing TSS, FAA and TPs contents. This is in parallel with previous findings in other plant species, such as bell pepper (*Capsicum annuum*) and common bean (*Phaseolus vulgaris*)^10,62^. TSS can also contribute to the regulation of osmotic pressure and expression of genes involved in metabolic processes, storage functions and salt-defense mechanisms^63^.

Pro accumulation is one of the most common alterations generated by high salt stress. However, it is debatable whether this accumulation is a mechanism of stress tolerance or merely a signal of the presence of stress^64^. Apart from its crucial role in osmoregulation, Pro over-accumulation alleviates salt stress by acting as a respiratory substrate to provide energy and help plants recover from stress^65^. In addition, production of Pro has been linked with scavenging some of the damaging ROS in order to reduce oxidative stress caused by salt stress^23,66^. In general, abiotic stresses leads to the accumulation of ROS and disturbance of redox homeostasis^67^. When plants face such challenges, they typically accumulate more antioxidant molecules such as phenolics, tocopherols and Car^68^. Our findings showed that salt-stressed fennel plants accumulated phenolic compounds in their dry leaves, particularly aglycon-phenolic, that are involved in ROS-scavenging and osmotic potential regulation.

Eventhough fennel plants are known for their high content of flavonoid compounds^69,70^, TFC of plants growing in saline calcareous soil dramatically decreased due to the high levels of ECe, CaCO_3_ and MgCO_3_^71^. LAB, especially those belonging to the genus *Lactobacillus*, can positively affect several important biomarkers in plants under stress^10,62^. In the current study, inoculated seeds with *Lp* and *Ll* and combined with either FM or PM amendments increased the levels of TFC in fennel plants, and consequently the antioxidant activity. This could probably be due to the breakdown of intricate polyphenol by enzymes existing during the lactic acid fermentation^72,73^.

Abiotic stresses cause partial stomatal closure, implying lower demand for ATP and reduction equivalents (NADP^+^) in Calvin cycle for CO_2_ fixation. As a result, the pool of NADP^+^ electron acceptors is depleted^74^. The biosynthesis of highly reduced specialized metabolites, such as monoterpenes (precursors of EO), alkaloids, aromatic amino acids and phenolics, aims at replenishing the pool of NADP^+^ through consumption of the reduced equivalents that cannot be directed to Calvin cycle^75^.

In the present investigation, the non-enzymatic antioxidants in fennel plants were shown to be considerably affected by soil organic amendments and/or LAB inoculation. Metabolic responses are highly dependent on the species and growth stage of plants, and the type and duration of stress^76^. AsA recycles free radicals to their reduced form, allowing reduction of additional H_2_O_2_ molecules^77,78^. Compared to stressed control plants, AsA and GSH content increased when organic fertilizers (FM or PM) were used alone or combined with *Lp* or *Ll* (Table 5). The combined effects of AsA and GSH, as a defense mechanism against oxidative stress, could explain the first increase in AsA. The two metabolites, AsA and GSH, are the main redox buffers in plant cells. They are often found in high concentrations in chloroplasts and other cellular compartments (5–20 mM AsA and 1–5 mM GSH)^79^.

The decrease in lipid peroxidation -represented by the cellular toxicity bioindicator MDA- can be attributed to the reduced levels of OH^−^, H_2_O_2_ and O_2_^−^ generation in the plant chloroplasts^59^. Our results also demonstrated that the yield components, such BY, SY, HI, SI and EOY of fennel plants, significantly decreased in saline calcareous soil. This is consistent with other reports that salinity stress can induce changes in pigment composition^80,81^, and SY and EOY content and composition^82,83^. Furthermore, organic manure (FM or PM) applied to saline calcareous soil, followed by sowing fennel seeds previously inoculated with a mixture of *Lp* + *Ll* considerably (*P* ≤ 0.05) augmented the average BY, SY, HI, SI and EOY (Table 6).

Results coming from the current study also showed that treatments with organic manure diminished the destructive effects in fennel plants grown in saline calcareous soil through the reduction in Na^+^ uptake and enhancement of K^+^ and Ca^2+^ uptake; thus, reducing osmotic stress and oxidative stress. In a parallel study by Kusvuran et al.^18^, they have observed increased oxidative stress by salt stress in sweet sorghum. Inoculation with *Lp* (ATCC 9019) decreased the antioxidant activity of *S. chirayita* plants, except of guaiacol peroxidase, when salt concentrations were elevated^56,84^.

LAB have proven to solubilize P as plant growth promoters^85,86^. FM or PM enhances soil fertility by increasing macronutrient availability, nutritional status and the population of soil microorganisms, and improving osmotic adjustment via osmolyte accumulation in stressed plants^61,87,88^. Na^+^ content in salt-stressed plants supplemented with LAB was lower than those in non-treated plants (Table 7). This suggests that LAB can exclude Na^+^ absorption by roots and prevent the translocation to shoot tissues. The reduction of Na^+^ accumulation and improvement of N, P, K^+^ and Ca^2+^ uptake could be a mitigation mechanism in stressed fennel plants supplemented by lactobacilli. This could further support the conclusion of a previous study^10^ that LAB (mainly lactobacilli) had positive effects on the development of the plant osmotic mechanism under salt stress conditions. 

### Supplementary Information

Supplementary Information.

## Data Availability

The data presented in this study are available upon request from the corresponding author.
