# Supplementary material for: Application of bio-organic amendments improves soil quality and yield of fennel (Foeniculum vulgare Mill.) plants in saline calcareous soil
Source: Sci Rep. 2023 Nov 14;13:19876. doi: 10.1038/s41598-023-45780-2 (PMC10646079; doi:10.1038/s41598-023-45780-2)
Supplement: Supplementary file 1 — Supplementary Information. [file 41598_2023_45780_MOESM1_ESM.pdf]

Supplementary Data

Application of bio-organic amendments improves soil quality and yield of fennel (*Foeniculum vulgare* Mill.) plants in saline calcareous soil

Omar A.A.I. Al-Elwany, Abir M.H.A. Mohamed, Ahmed S. Abdelbaky, Mohamed A. Tammam, Khaulood A. Hemida, Gehad H.S. Hassan, Mohamed T. El-Saadony, Khaled A. El-Tarabily*, Synan F. AbuQamar*, Taia A. Abd El-

*** Correspondence:**

Prof. Khaled El-Tarabily: ktarabily@uaeu.ac.ae

Prof. Synan AbuQamar: sabuqamar@uaeu.ac.ae

Table S1. Chemical properties of FM and PM.

Properties	Unit	FM	PM
pH		7.56	7.42
ECe	dS m^{-1}	3.98	4.50
OM	%	39.5	45.6
N		1.16	1.36
P		0.57	0.49
K		1.89	1.57

FM, farmyard manure; PM, poultry manure; ECe, electrical conductivity of saturated soil extract; OM, organic matter; N, nitrogen; P, phosphorus; K, potassium.

Table S2. Morphological, physiological, and biochemical characters of LAB.

<table>
<tr><th>Characteristic</th><th>Lactobacillus plantarum</th><th>Lactococcus lactis</th></tr>
<tr><td>Gram stain reaction</td><td colspan="2">+</td></tr>
<tr><td>Motility</td><td colspan="2">–</td></tr>
<tr><td>Spore formation</td><td colspan="2">–</td></tr>
<tr><td>Oxygen utilization</td><td colspan="2">Facultative</td></tr>
<tr><td>Growth at 8-40°C</td><td colspan="2">+</td></tr>
<tr><td>Hormonal production</td><td colspan="2"></td></tr>
<tr><td>Indole</td><td colspan="2">+</td></tr>
<tr><td>Cytokinin</td><td>+</td><td>–</td></tr>
<tr><td>Gibberellic acid</td><td>+</td><td>–</td></tr>
<tr><td>Citrate utilization</td><td colspan="2">+</td></tr>
<tr><td>Carbohydrates fermentation</td><td colspan="2"></td></tr>
<tr><td>D-glucose</td><td colspan="2">+</td></tr>
<tr><td>Lactose</td><td colspan="2">+</td></tr>
<tr><td>D-mannitol</td><td colspan="2">+</td></tr>
<tr><td>D-xylose</td><td colspan="2">+</td></tr>
<tr><td>Production of organic acids</td><td colspan="2"></td></tr>
<tr><td>Lactic</td><td colspan="2">+</td></tr>
<tr><td>Citric</td><td colspan="2">+</td></tr>
<tr><td>Tartaric</td><td colspan="2">+</td></tr>
<tr><td>Malic</td><td colspan="2">+</td></tr>
<tr><td>Gluconic</td><td colspan="2">+</td></tr>
<tr><td>Acetic</td><td colspan="2">+</td></tr>
<tr><td>Osmolarity; growth at</td><td colspan="2"></td></tr>
<tr><td>5%</td><td colspan="2">+</td></tr>
<tr><td>10%</td><td colspan="2">+</td></tr>
<tr><td>15%</td><td colspan="2">–</td></tr>
<tr><td>pH; growth at 4.4</td><td colspan="2">+</td></tr>
<tr><td>pH; growth at 9.6</td><td colspan="2">–</td></tr>
<tr><td>Production of metabolites and hydrolytic enzymes</td><td colspan="2"></td></tr>
<tr><td>Oxidase</td><td>–</td><td>+</td></tr>
<tr><td>Catalase</td><td colspan="2">–</td></tr>
<tr><td>Nitrite reductases</td><td>+</td><td>–</td></tr>
<tr><td>Gelatin hydrolysis</td><td colspan="2">+</td></tr>
<tr><td>Starch hydrolysis</td><td colspan="2">+</td></tr>
<tr><td>Cellulose hydrolysis</td><td colspan="2">+</td></tr>
<tr><td>Chitinase</td><td colspan="2">+</td></tr>
<tr><td>Protease</td><td colspan="2">+</td></tr>
<tr><td>Lipase</td><td colspan="2">–</td></tr>
<tr><td>PGP activities</td><td colspan="2"></td></tr>
<tr><td>Siderophores production</td><td colspan="2">+</td></tr>
<tr><td>Polyphenols production</td><td colspan="2">+</td></tr>
<tr><td>Phosphate solubilization</td><td colspan="2">+</td></tr>
<tr><td>Putative nitrogen fixation</td><td colspan="2">–</td></tr>
<tr><td>Antifungal activity</td><td colspan="2"></td></tr>
<tr><td>Fusarium oxysporum</td><td colspan="2">+</td></tr>
<tr><td>Phytophthora infestans</td><td>+</td><td>–</td></tr>
<tr><td>Botrytis cinerea</td><td colspan="2">+</td></tr>
</table>

Penicillium sp.	+

LAB, lactic acid bacteria, PGP, plant growth promoting; +, present/producing; -, absent/not producing.
